# Dynamic single-slice CT estimates whole-lung dual-energy CT variables in pigs with and without experimental lung injury

**DOI:** 10.1186/s40635-019-0273-y

**Published:** 2019-11-01

**Authors:** John N. Cronin, João Batista Borges, Douglas C. Crockett, Andrew D. Farmery, Göran Hedenstierna, Anders Larsson, Minh C. Tran, Luigi Camporota, Federico Formenti

**Affiliations:** 1 Centre for Human and Applied Physiological Sciences, King’s College London, London, UK; 2 Nuffield Division of Anaesthetics, University of Oxford, Oxford, UK; 3 Hedenstierna Laboratory, Department of Medical Sciences, Uppsala University, Uppsala, Sweden; 4 Hedenstierna Laboratory, Department of Surgical Sciences, Uppsala University, Uppsala, Sweden; 5 Department of Adult Critical Care, St. Thomas’ Hospital, Guy’s and St. Thomas’ NHS Foundation Trust, King’s Health Partners, London, UK

**Keywords:** Tomography, X-ray computed, Respiration, Artificial, Contrast media, Acute lung injury, Swine

## Abstract

**Background:**

Dynamic single-slice CT (dCT) is increasingly used to examine the intra-tidal, physiological variation in aeration and lung density in experimental lung injury. The ability of dCT to predict whole-lung values is unclear, especially for dual-energy CT (DECT) variables. Additionally, the effect of inspiration-related lung movement on CT variables has not yet been quantified.

**Methods:**

Eight domestic pigs were studied under general anaesthesia, including four following saline-lavage surfactant depletion (lung injury model). DECT, dCT and whole-lung images were collected at 12 ventilatory settings. Whole-lung single energy scans images were collected during expiratory and inspiratory apnoeas at positive end-expiratory pressures from 0 to 20 cmH_2_O. Means and distributions of CT variables were calculated for both dCT and whole-lung images. The cranio-caudal displacement of the anatomical slice was measured from whole-lung images.

**Results:**

Mean CT density and volume fractions of soft tissue, gas, iodinated blood, atelectasis, poor aeration, normal aeration and overdistension correlated between dCT and the whole lung (*r*^2^ 0.75–0.94) with agreement between CT density distributions (*r* 0.89–0.97). Inspiration increased the matching between dCT and whole-lung values and was associated with a movement of 32% (SD 15%) of the imaged slice out of the scanner field-of-view. This effect introduced an artefactual increase in dCT mean CT density during inspiration, opposite to that caused by the underlying physiology.

**Conclusions:**

Overall, dCT closely approximates whole-lung aeration and density. This approximation is improved by inspiration where a decrease in CT density and atelectasis can be interpreted as physiological rather than artefactual.

## Background

Intra-tidal changes in lung aeration and atelectasis are common in animal models of the acute respiratory distress syndrome (ARDS) [1, 2]. Quantifying such intra-tidal events is important as cyclical atelectasis remains one of the key pathophysiological mechanisms underlying ventilator-induced lung injury (VILI) [3]. Most studies examining these rapid changes have used a ‘single-slice’ dynamic CT (dCT) approach: a fixed CT scanner field-of-view images a slice of the thorax over time, whilst the lung moves during ventilation [1, 4–7]. This approach allows sufficient temporal resolution to study intra-tidal changes, but is blind to what occurs outside the field-of-view. Two main questions associated with the dCT approach arise: (1) is the single-slice representative of the pathophysiological behaviour of the rest of the lung and (2) is the movement of the lung during ventilation sufficient to cause different regions of the lung to be imaged during inspiration and expiration, thus reducing the validity of comparisons between these two conditions?

Human patients with ARDS exhibited no cranio-caudal gradient in CT lung densities [8]; thus, it would be expected that an appropriately chosen slice of the lung perpendicular to the cranio-caudal axis could be representative of the entire lung. However, as the lung expands caudally with flattening of the diaphragm during inspiration, this relationship may not remain constant during the ventilatory cycle. In particular, as the regions around the hila contain a larger volume of both blood within major vessels and air within major bronchi, an artificial inspiratory-related increase in both atelectatic and hyperinflated regions may be expected.

Several studies have investigated these issues. In healthy pigs, a single dCT slice 3 cm caudal to the carina overestimated atelectatic volume within the lung by 28.4% compared with 19.0% in whole lung volume scans taken during inspiratory apnoeas [9]. Following induction of a lung injury model, dCT slices in expiration or inspiration showed distributions of CT densities similar to the same regions imaged during breath holds at end-expiration or end-inspiration, respectively [10]. Additionally, large variations were not observed in the composition of the neighbouring anatomical slices from the slice, where the dCT image was taken, although these results were from static scans taken during breath holds. Both studies used non-contrast CT scans however, so it is impossible to quantify the impact that large blood vessels moving into the scan field would have had upon CT density distributions.

We sought to further demonstrate the appropriateness of the single-slice dCT technique in predicting whole-lung values. First, we aimed to replicate the results of the above experiments and comprehensively extend them to include results following three-material differentiation of dual-energy CT (DECT) images, which are being more commonly used to quantify blood volume within the lung [11, 12], and would allow separation of blood vessel artefact from true atelectasis. This technology images the same region of the lung at two separate X-ray energies and is able to determine the relative contributions of three separate ‘materials’ to each voxel. These materials are typically tissue types (e. g. soft tissue, bone or fat) or other materials such as air or iodine contrast agent. As for single-energy CT, DECT can also be employed in dCT and whole-lung modes, and the corresponding single-energy images can be readily reconstituted from the DECT images [13–15]; thus, any validation for DECT images also holds for single-energy CT.

We also investigated the change in imaged lung parenchyma that occurs with an inspiratory breath by comparing dCT and whole-lung measurements of various CT indices (density, atelectasis, etc.) throughout the respiratory cycle. Finally, we aimed to use automated morphological analysis to generate a three-dimensional model of the regional strain within the lung and used this model to determine the inspiration-related displacement of selected dCT slices in the cranio-caudal direction.

## Methods

This study received ethical approval (Uppsala Regional Animal Research Ethics Committee ref. C98/16) and conformed with the relevant sections of the ARRIVE guidelines [16]. Eight domestic pigs were studied under general anaesthesia. Full details of the anaesthesia and instrumentation are available in Additional file 1.

### Lung injury model

In four randomly chosen animals, a saline-lavage surfactant-depletion lung injury model was created using the technique of Lachmann [17] aiming for a PaO_2_/FiO_2_ (P/F) ratio of 150–200 mmHg measured at a positive end-expiratory pressure (PEEP) level of 5 cmH_2_O and FiO_2_ of 0.7. If required, an infusion of noradrenaline (0.01 to 0.1 mcg/kg/min) was commenced following injury to maintain mean arterial blood pressure greater than 65 mmHg.

### CT scan sequence

**Fig. 1:**
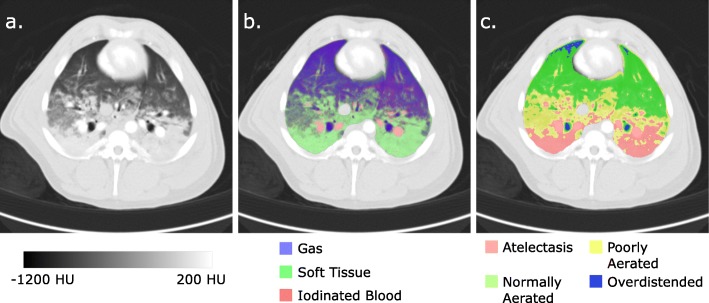
Example CT images of the juxtadiaphragmatic slice used in this study. **a** 70%:30% merge of the two dual-energy CT images using standard lung windows. **b** Results of segmentation to exclude extrapulmonary structures and dual-energy three material differentiation demonstrating gas, soft tissue and iodinated blood volume fractions. **c** Segmentation to identify regions of overdistension (gas volume fraction > 90%), normal aeration (gas volume fraction 50–90%), poor aeration (gas volume fraction 10–50%) and atelectasis (gas volume fraction < 10%). *HU* Hounsfield units

All CT images were collected with a Somatom Definition Flash dual-source CT scanner (Siemens, Erlangen, Germany). First, single-energy volume scans at PEEP levels from 0 to 20 cmH_2_O were collected during apnoeas at end-inspiration and end-expiration for the morphological analysis technique. After this, 12 different ventilatory conditions (combinations of three tidal volumes (*V*_*T*_) from 7 to 15 mL/kg and four PEEP settings from 5 to 12 cmH_2_O) were studied in a random order and 1 Hz contrast-enhanced DECT dCT images collected along with corresponding whole-lung DECT scans during both end-inspiratory and end-expiratory apnoeas. The slice chosen for dCT analysis was mid-way between the carina and diaphragm and an example is provided in Fig. 1a. A full description of the scanning sequence and slice selection is available in Additional file 1.

### DECT image processing

DECT volumes (for both dCT and static series) were initially processed using a custom three-material differentiation algorithm (slicerdect: 10.5281/zenodo.3346937) to determine the volume fractions of soft tissue, iodinated blood, and gas within each voxel. Example results of this algorithm are presented in Fig. 1b. DECT coefficients (i. e. the CT densities equivalent to 100% of each material at each energy level) were chosen as (1) the mean density of the descending thoracic aorta for iodinated blood, (2) the mean density of the parasternal musculature for soft tissue and (3) − 1000 HU at both energy levels for gas. Additionally, a 70%:30% merge volume from the two DECT volumes was created to be used for segmentation purposes.

Volume segmentation was similar for both dynamic and spiral CT images: lung parenchyma was included, with the exclusion of the heart, mediastinal contents (aorta, oesophagus and azygos vein) and inferior vena cava. The division into atelectatic, poorly aerated, normally aerated and overdistended regions was performed using the definitions as previously described [18]; however, the DECT gas volume fraction image was used instead of the source images to prevent overestimation of the atelectatic fraction due to iodine administration. As such, cut-offs were gas volume fractions of 10%, 50% and 90% (equivalent to − 100, − 500 and − 900 HU, respectively, on non-contrast scans), and an example is provided in Fig. 1c.

dCT frames were divided into inspiratory or expiratory based upon the mean gas volume (slice volume multiplied by mean gas volume fraction) within each frame. For each ventilatory condition in each animal, the frames containing the maximum and minimum gas volumes were identified. Frames where gas volumes lay within 30% of the minimum or maximum value were deemed to be expiratory or inspiratory, respectively. Other frames were excluded from the analysis.

Distributions of merge volume voxels (counted within 10 HU-wide bins from − 1000 to + 500 HU) and soft tissue, gas and iodinated blood volume fractions (within 1% wide bins from 0% to 100%) were determined for each ventilatory condition from both dCT and whole-lung imaging. Counts were normalised such that each histogram summed to 1 to allow comparison between dCT and whole-lung images that contained differing numbers of voxels.

### Morphological analysis

High spatial resolution single-energy images taken during expiratory breath holds were registered onto the corresponding inspiratory images using the NiftyReg software [19] which has previously been used to assess regional strain in the lung [20, 21]. Briefly, this software attempts to minimise the difference between the moving (expiratory) and fixed (inspiratory) images by optimising the fitting of a B-spline free form deformation matrix. This matrix describes how much each point within a three-dimensional grid with 3 × 3 ×  3 mm spacing moves between the expiratory and inspiratory images, with smoothed curves between these points used to estimate the actual displacement. The accuracy of the registration was assessed manually and then a single 5 mm slice, equivalent to that used in the dCT images, was segmented and divided into 12 regions from anterior to posterior (Fig. 5b). The mean cranio-caudal displacement of each region (i. e. the component of the displacement vector for each voxel in the cranio-caudal direction that is required to make the expiratory volume look similar to the inspiratory volume) for each PEEP setting in each animal was calculated.

### Statistical analyses

Eight variables of interest were chosen to determine the agreement between dCT and whole-lung images. These were CT density of the merged volume, and volume fractions of soft tissue, gas, iodinated blood as well as atelectatic, poorly aerated, normally aerated and overdistended regions. Mean values of each of these variables between dCT and whole-lung values were investigated with linear correlation, Bland-Altman analysis and paired *t* test. Correlation between distributions of merge volume CT densities as well as soft tissue, gas and iodinated blood volume fractions was assessed using Pearson’s product-moment correlation coefficient. To investigate the effect of cranio-caudal position within the three concurrently acquired dCT slices, the mean variation in the middle and cranial slice compared with the caudal slice was determined for each variable of interest and linear regression employed to determine the effect of a 1-mm change in position upon each particular variable. Further details of statistical handling are provided in Additional file 1.

## Results

All eight animals completed both the combined expiratory/inspiratory spiral CT volume scans for morphological analyses at each of 5 PEEP levels from 0 to 20 cmH_2_O and the 12 different ventilatory conditions (PEEP 5–12 cmH_2_O, *V*_*T*_ 7–15 mL/kg) used for the comparison between dCT and whole-lung DECT. Baseline characteristics of the animals are provided in Table 1, with a reduction in P/F ratio to 179 mmHg (interquartile range 88 mmHg) and static compliance to 19.9 (3.8) mL/cmH_2_O seen in the injured-lung group.

**Table 1:** Baseline characteristics of the uninjured and injured groups

	Uninjured	Injured
*n*	4	4
Ventilatory conditions per animal	12	12
Male:female	3:1	4:0
Weight (kg)	31.3 (2.6)	30.8 (1.35)
Lavage volume (mL/kg)	N/A	95 (97)
PaO_2_/FiO_2_ ratio (mmHg)	350 (48.2)	179 (88)
Static compliance (mL/cmH_2_O)	28.0 (8.3)	19.9 (3.8)
Cardiac output (L/min)	4.8 (1.8)	4.8 (0.3)
Oxygen consumption (mL/min)	166 (96)	228 (31)

Data expressed as median (interquartile range) where appropriate. PaO_2_/FiO_2_ ratio and static compliance were measured at PEEP 5 cmH_2_O

### Mean CT density and volume fractional differences between dCT and whole-lung DECT

**Fig. 2:**
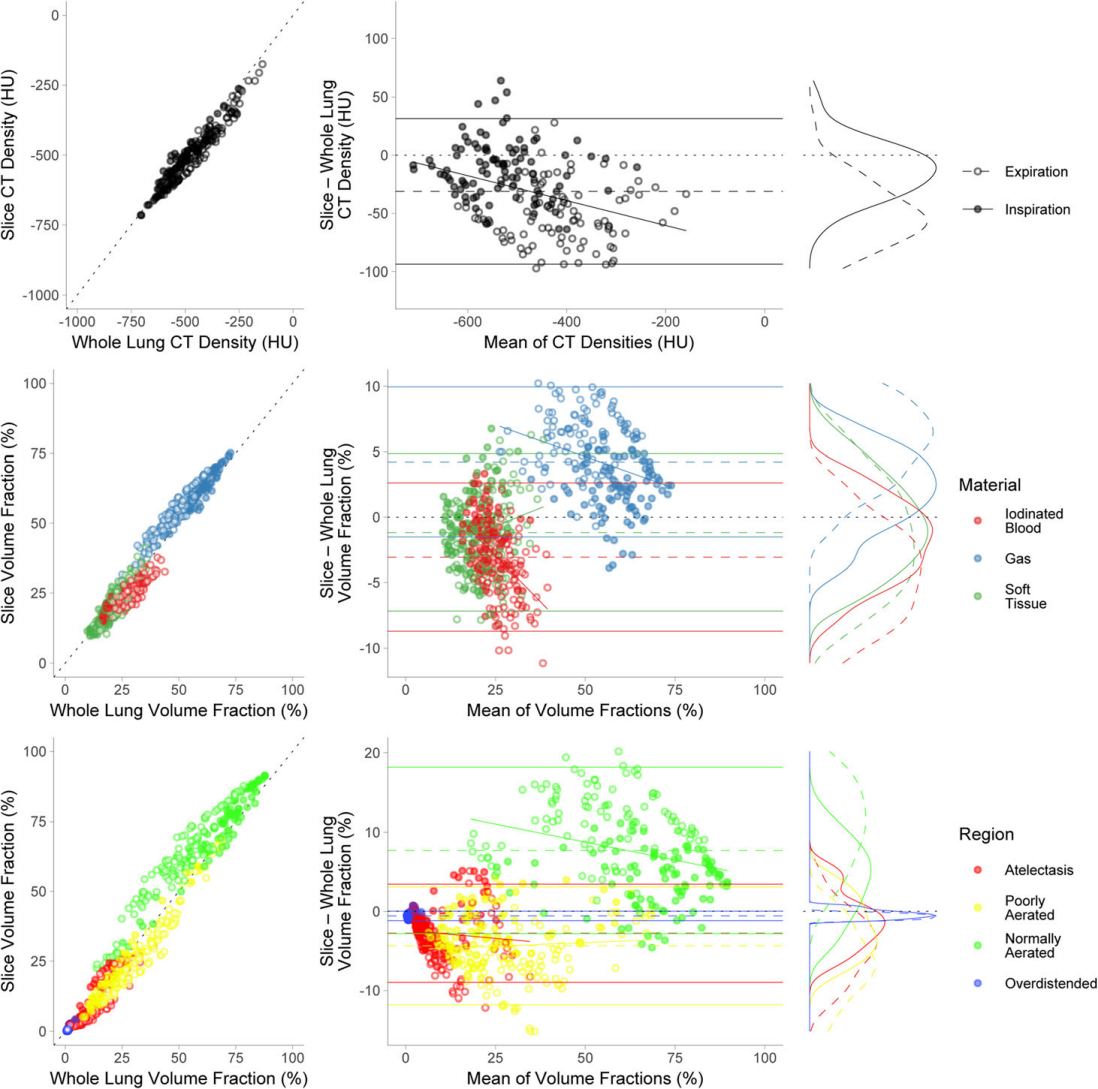
Correlation (left column) and Bland-Altman analysis (middle column) between dynamic CT and whole-lung CT means of eight variables studied. Black dotted line is the identity line in the correlation graphs and the line of no difference in the Bland-Altman analysis. Mean difference is represented by dashed line with solid lines representing 95% limits of agreement in the Bland-Altman analysis. The greatest effect on the discrepancy between dynamic CT and whole-lung values was noted between inspiration (filled circles) and expiration (empty circles): thus, these points are highlighted separately. Right-hand column provides density distributions for the discrepancies, again separated by inspiration (solid line) and expiration (dashed line). In all eight variables, inspiration moves the distribution closer to the line of no difference

Single-slice dCT correlated with whole-lung DECT measures for mean CT density and soft tissue, gas, iodinated blood, atelectatic, poorly aerated, normally aerated and overdistended volume fractions (Fig. 2). Relevant correlation coefficients, mean differences and limits of agreement are summarised in Table 2. Overall, dCT underestimated CT density and volume fractions of soft tissue, iodinated blood, atelectasis, poor aeration and overdistension, and overestimated volume fractions of gas and normal aeration. The greatest effect upon this bias was seen with inspiration, which reduced the discrepancy between dCT and whole-lung values for each of the eight parameters measured (Fig. 2, marginal histograms; Table 3). The effects of PEEP, *V*_*T*_ and injury were minimal and are provided in Additional file 1.

**Table 2:** Comparison of means of dCT and single-slice variables

Variable	*r* ^2^	Mean difference	Lower 95% limit of agreement	Upper 95% limit of agreement
CT density (HU)	0.93	− 31.2 [− 35.7 to − 26.6]	− 93.8 [− 101.6 to − 86.0]	31.5 [23.7 to 39.3]
FV-soft tissue (%)	0.78	− 1.2 [− 1.6 to − 0.7]	− 7.2 [− 7.9 to − 6.4]	4.8 [4.1 to 5.6]
FV-gas (%)	0.92	4.2 [3.8 to 4.6]	− 1.5 [− 2.2 to − 0.8]	10.0 [9.2 to 10.7]
FV-iodinated blood (%)	0.75	− 3.1 [− 3.5 to − 2.6]	− 8.7 [− 9.4 to − 8.0]	2.6 [1.9 to 3.3]
FV-atelectasis (%)	0.83	− 2.8 [− 3.2 to − 2.3]	− 9.0 [− 9.7 to − 8.2]	3.4 [2.7 to 4.2]
FV-poorly aerated (%)	0.94	− 4.4 [− 4.9 to − 3.8]	− 11.8 [− 12.7 to − 10.9]	3.1 [2.1 to 4.0]
FV-normally aerated (%)	0.92	7.7 [6.9 to 8.5]	− 2.8 [− 4.1 to − 1.5]	18.2 [16.9 to 19.5]
FV-overdistended (%)	0.80	− 0.6 [− 0.6 to − 0.5]	− 1.2 [− 1.2 to − 1.1]	0.0 [− 0.0 to 0.1]

Correlation and Bland-Altman analysis of agreement for the means of eight different variables between dynamic single-slice and whole-lung dual-energy CT. Dynamic single-slice and whole-lung measurements were different for all variables measured (all *P* < 0.0001). Mean difference and limits of agreement expressed as mean [95% confidence interval]. *n* = 96 ventilatory conditions across 8 animals. *FV* volume fraction, *HU* Hounsfield units

**Table 3:** Limits of variable bias due to inspiration between dCT and whole-lung means

Variable	Expiratory bias	Inspiratory bias	Difference	*P*
CT density (HU)	− 51 (26)	− 12 (24)	39.08 [11.35 to 66.8]	< 0.001
FV-soft tissue (%)	− 1.7 (3.4)	− 0.7 (2.7)	0.99 [− 1.48 to 3.45]	0.013
FV-gas (%)	6.1 (2.4)	2.4 (2.2)	− 3.66 [− 6.23 to − 1.1]	< 0.001
FV-iodinated blood (%)	− 4.4 (2.9)	− 1.7 (2.3)	2.67 [0.61 to 4.73]	< 0.001
FV-atelectasis (%)	4.1 (3.2)	− 1.4 (2.5)	2.67 [0.00 to 5.33]	< 0.001
FV-poorly aerated (%)	− 6.4 (3.5)	− 2.3 (2.9)	4.05 [− 0.68 to 8.79]	< 0.001
FV-normally aerated (%)	11.1 (4.4)	4.2 (3.8)	− 6.92 [− 12.11 to − 1.72]	< 0.001
FV-overdistended (%)	− 0.7 (0.2)	− 0.5 (0.3)	0.19 [− 0.29 to 0.68]	< 0.001

Expiratory and inspiratory bias values presented as mean (SD) and difference as mean [95% limits, i.e. ± 1.96xSD]. *FV* volume fraction; *HU* Hounsfield units

### Correlation between CT density and volume fraction distributions

**Fig. 3:**
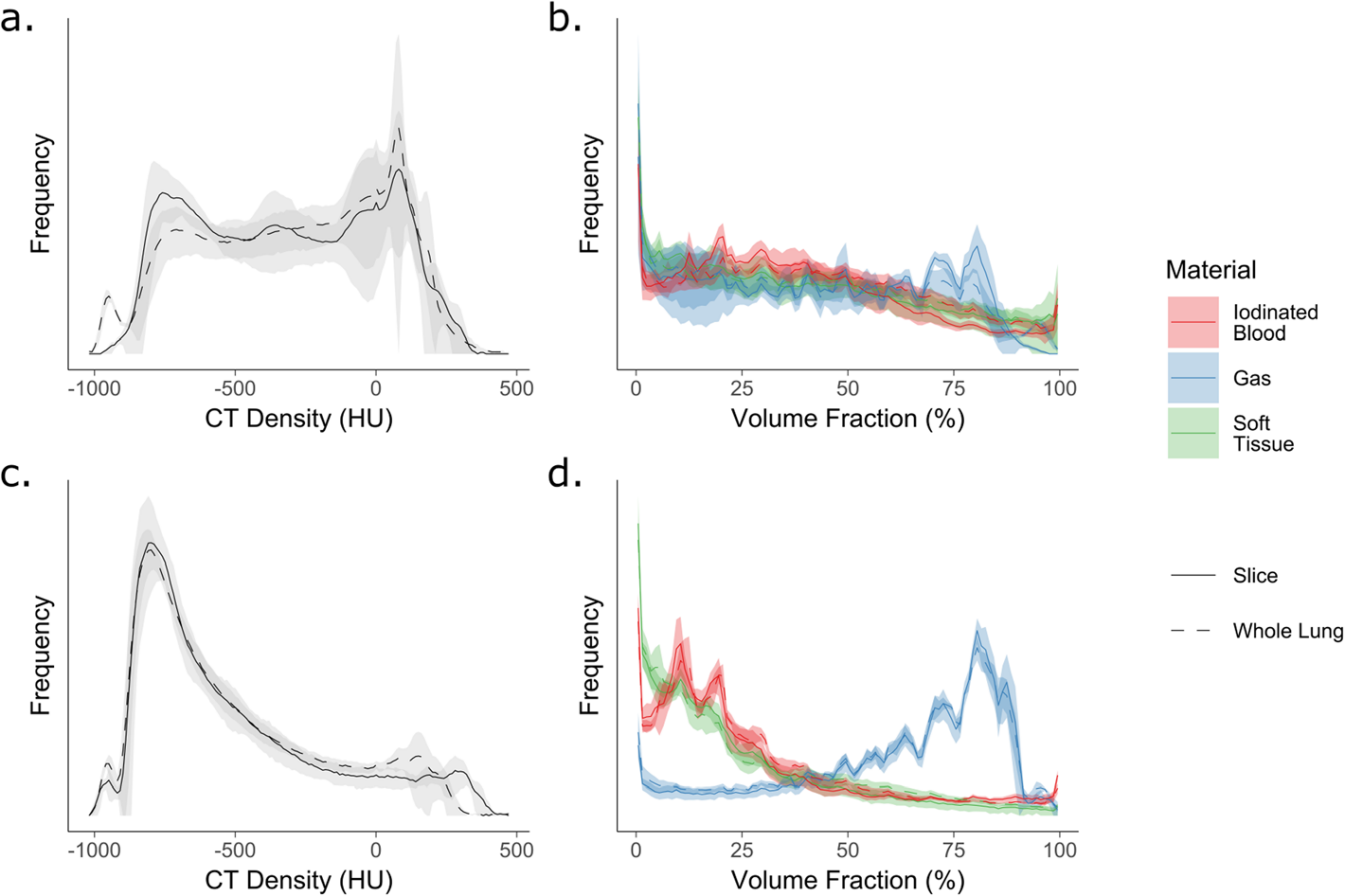
Example distributions of CT densities (**a**, **c**) and those of the results of the three-material differentiation (**b**, **d**) between dynamic CT (solid line) and whole lung (dashed line). **a**, **b** PEEP 5 cmH_2_O, tidal volume 7 mL/kg, injured animals in expiration. **c**, **d** PEEP 12 cmH_2_O, tidal volume 15 mL/kg, uninjured animals in inspiration. There is an increased correlation between the two distributions in inspiration at higher PEEP and tidal volumes. Lines and shaded areas represent mean and standard deviations of *n* = 4 animals in each group

Overall, correlation between dCT and whole-lung distributions was good: *r* = 0.93 (0.10), 0.97 (0.03), 0.89 (0.11) and 0.91 (0.09) for CT density and soft-tissue, gas and iodinated blood volume fractions, respectively; data expressed as median (IQR). The greatest change in correlation was seen with the effect of inspiration for gas volume fractions (0.84 (0.16) to 0.93 (0.07); *P* < 0.001) and iodinated blood volume fractions (0.88 (0.11) to 0.94 (0.06); *P* < 0.001). Example distributions are provided in Fig. 3 and the results for effects of injury, PEEP and *V*_*T*_ provided in Additional file 1: Table S2.

### Differences between three adjacent dCT slices

**Fig. 4:**
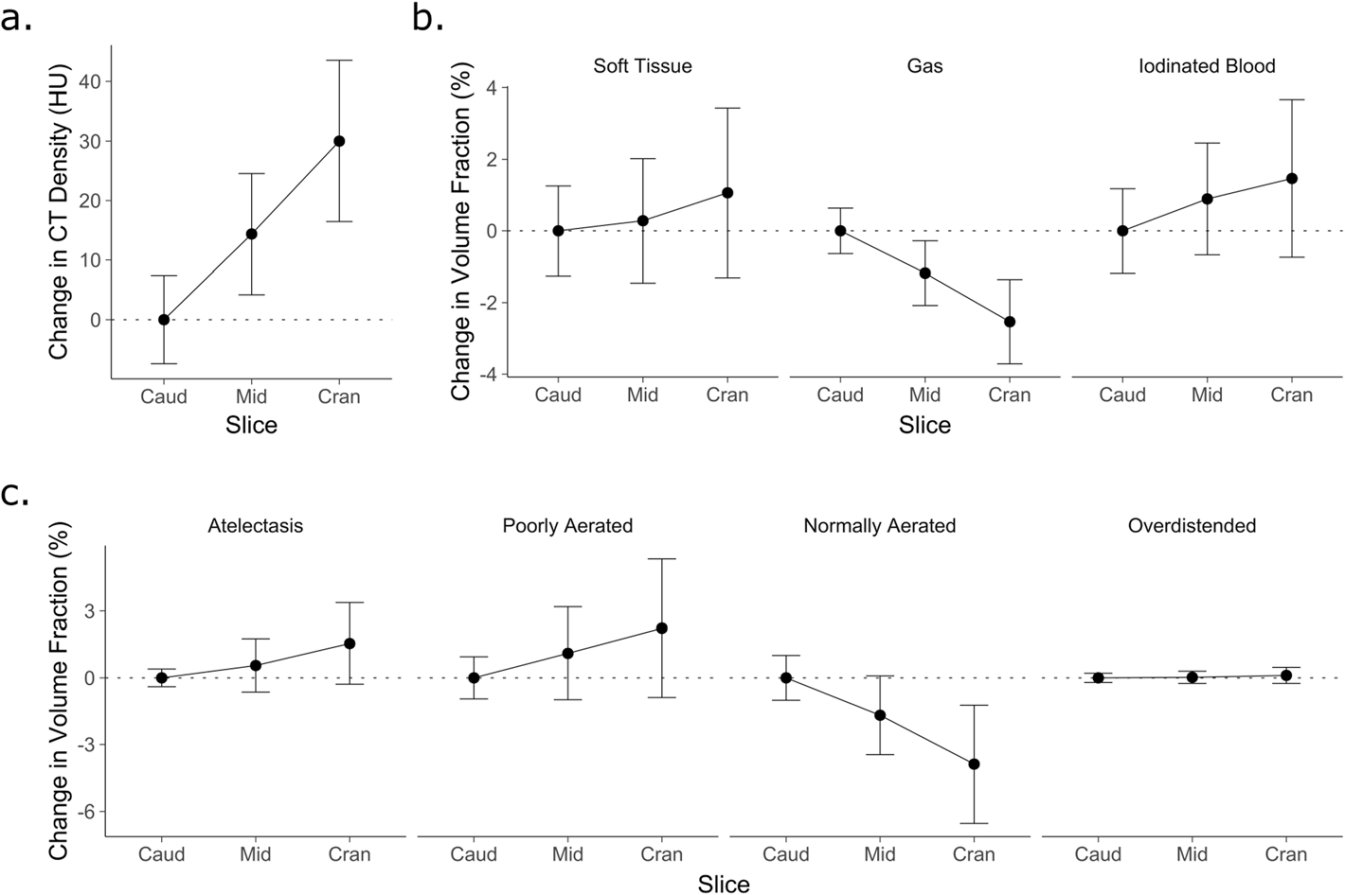
Variation in the means of eight studied variables between three adjacent 5-mm juxtadiaphragmatic dynamic CT slices acquired concurrently. Values are presented relative to the mean value in the most caudal slice. **a** Mean slice density, **b** results of the three-material differentiation and **c** following segmentation based upon gas density. A good correlation was seen between cranio-caudal position and CT density, gas and normally aerated volume fractions. Points and error bars represent mean and SD of 13–16 frames (dependent on exclusion of frames mid-way between expiration and inspiration) in 12 ventilatory conditions in 8 animals. *Caud* caudal slice, *Mid* middle slice, *Cran* cranial slice

Mean CT density in the three adjacent slices was positively correlated with cranial distance (*r*^2^ = 0.80). Each 1-mm movement in the cranial direction was associated with an increase in CT density of 3.0 HU. Gas volume fraction decreased by 0.3%/mm (*r*^2^ = 0.78) and normally aerated volume fraction decreased by 0.4%/mm (*r*^2^ = 0.62). Whilst reciprocal trends in other variables were seen (Fig. 4), the correlation was minimal (all *r*^2^ < 0.3).

### Morphological analysis of effects of inspiration on whole-lung spiral CT volumes

**Fig. 5:**
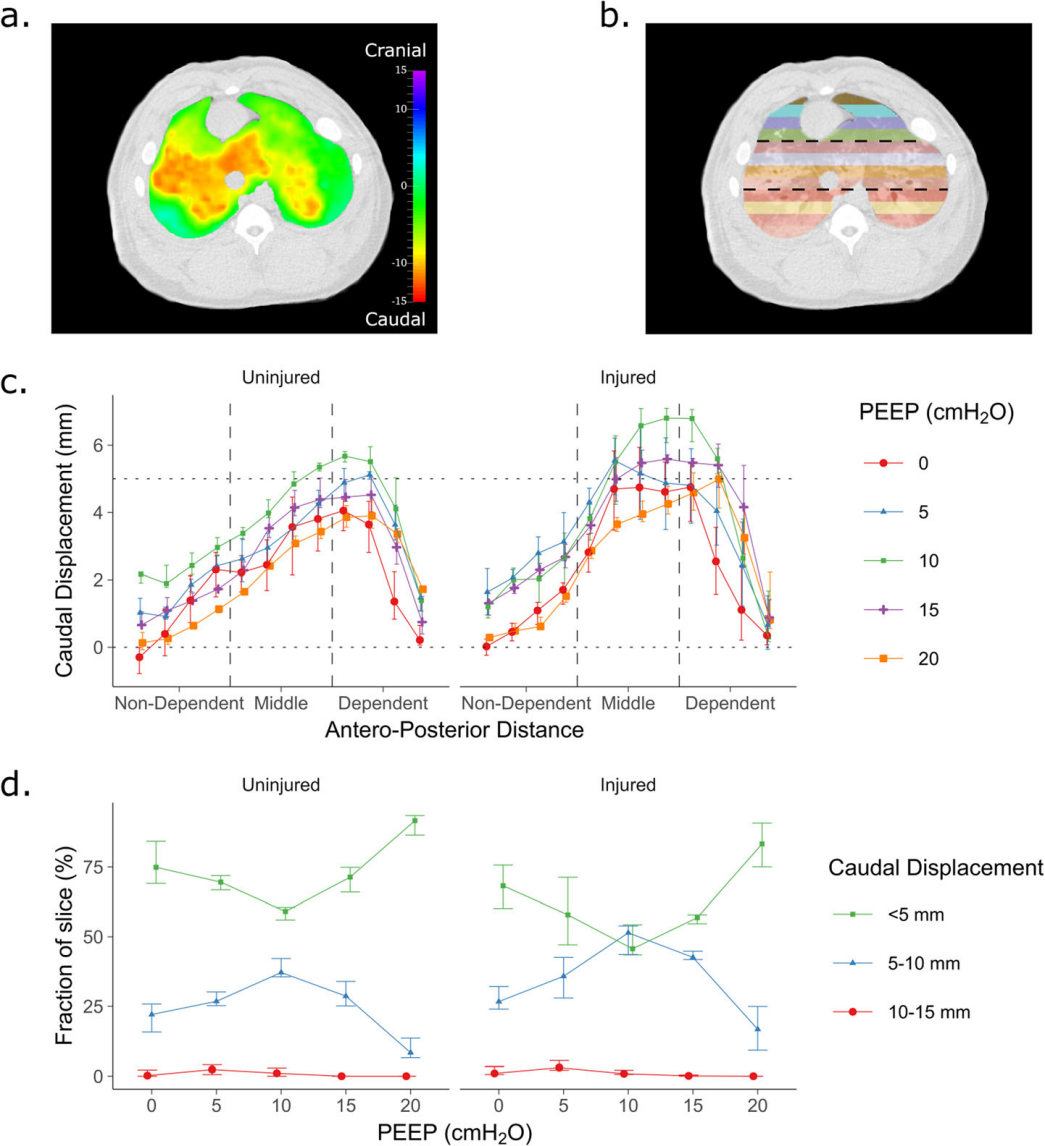
Results of the morphological analysis of single-energy volume CT scans between expiration and inspiration. **a** Example of the level of the whole-lung CT equivalent to that studied for the dynamic CT series, taken from an injured animal at PEEP 10 cmH_2_O. Overlaid is the cranio-caudal displacement of individual voxels in the slice demonstrating the majority of the displacement occurring in the middle third of the lung along the gravitational vector. **b** Example segmentation into 12 regions of equal height along the gravitational vector. **c** Quantification of the caudal displacement of the slice between expiration and inspiration in each of the 12 regions at different PEEP levels. Division into non-dependent, middle and dependent regions as per thick horizontal dashed lines in **b**. **d** The fraction of the slice that moves less than one slice (< 5 mm), between one and two slices (5–10 mm) and more than 2 slices (10–15 mm) in inspiration when grouped by PEEP. Points in **c** and **d** represent median and IQR of 4 injured and 4 uninjured animals

Visual inspection of the deformation matrix applied to the moving (expiration) volume confirmed its similarity to the fixed (inspiration) volume in all cases. At the level of the studied slice, no voxels moved by more than 5 mm in the cranial direction or 15 mm in the caudal direction. The greatest caudal displacement occurred in the middle third of the lung in the injured animals, and in the middle and dependent thirds in the uninjured animals (Fig. 5a, c).

On average, 32% (15%) (mean (SD)) of the slice moved more than 5 mm in the caudal direction with inspiration and in no cases was this fraction more than 58%. The fraction that moved more than 10 mm was minimal (1.6% (3.0%)). The fraction that moved more than 5 mm was greatest at PEEP 10 cmH_2_O in both injured and uninjured animals (*P* = 0.026 vs 0 cmH_2_O and < 0.0001 vs 20 cmH_2_O; Fig. 5d).

## Discussion

We showed that dynamic single-slice dual-energy CT images closely approximate whole-lung scans in pigs with and without a lung injury model over a variety of different PEEP levels and tidal volumes, with a small effect of inspiration upon this agreement. During inspiration, approximately one third of the slice moved out of the CT scanner field-of-view; however, it was replaced by the adjacent slice, which was of similar composition. The intra-tidal variation in bias between dCT and whole-lung imaging was less than 10% of the range of the studied parameter.

### Comparison of dCT images with those taken during breath-holds

Mean dCT variables closely correlated with those from whole-lung scans with *r*^2^ ≥ 0.75. There was a bias toward underestimating CT lung density, soft tissue density, iodinated blood density, atelectasis and areas of poor aeration on the dCT slices; however, the mean bias was 31.2 HU in terms of CT density (equivalent to 3% of the HU range from − 1000 to 0) and at most 7.7% of the volume fractions measured. The correlation coefficients between the 4 continuous distributions of voxels tested were all ≥ 0.89, consistent with excellent agreement. This latter result is also important because the mean voxel value seen in the dCT slice does not adequately describe the range of voxel densities with that slice—typically, a bimodal distribution was seen (Fig. 3). Taken together, these results provide strong evidence that the make-up of the single dCT slice chosen approximated the whole lung.

Previous experiments in uninjured pigs have demonstrated that a subcarinal slice overestimates both overdistension and atelectasis and underestimates ventilated regions [9], in contrast with our results. Interestingly, the overdistended regions represented a significantly smaller fraction of the slice/lung volume in our study compared with those previously described (0.6% in dCT vs 1.2% in the whole lung as compared with 6.4% in dCT vs 3.1% in the whole lung in published results [9]). A possible reason for this discrepancy is that our study used 5 mm instead of 1-mm-thick CT slices. When thicker slices are used, as in our study, overdistended regions may comprise less of the whole lung volume, presumably due to the heterogeneity within a larger voxel tending to be averaged toward an intermediate CT density [22, 23].

A separate study in an oleic-acid lung injury model in pigs has demonstrated no significant difference between either a juxtadiaphragmatic or apical dCT slice and that of the whole lung in terms of CT density distributions [10]. Examination of the density distributions in these results demonstrates a trend for dCT to underestimate atelectatic fraction in expiration. Despite the differences in the lung injury model, the volume fractions of atelectasis, poor aeration, normal aeration and overdistension in this study were similar to those we demonstrated.

### Effects of inspiration upon the imaged slice

Dynamic CT studies examine the effect of the ventilatory cycle upon the lung. As such, a bias between dCT and the whole-lung imaging may not be a major limitation: as long as this bias remains constant throughout the respiratory cycle, the delta-change values seen in dCT can be used to predict changes at the whole-lung level. We did, however, demonstrate a variable bias, which decreased in inspiration (Fig. 2, Table 3). This result suggests that the usage of dCT to estimate whole-lung CT parameters introduces an artefact, which could be erroneously identified as intra-tidal changes occurring within the lung. The mean variation in bias throughout the respiratory cycle was 39 HU in terms of CT density (equivalent to 3.9% of the typically investigated HU range between − 1000 and 0) and ranged between 0.1% and 6.9% for volume fractions of the various materials. We also report the 95% limits of these values such that researchers who demonstrate an effect size outside this range can be confident that the results seen are due to changes in the underlying pathophysiology, rather than as an artefact due to the use of dCT technology.

The direction of this variable bias is also important. In the three variables that demonstrated the largest artefactual effect of inspiration (CT density, poorly and normally aerated volume fractions), the variation in bias during inspiration is in the opposite direction to what would be expected. For example, normally aerated volume fraction typically increases with inspiration [7]; however, we demonstrated an artefactual reduction in bias associated with inspiration with 95% limits between − 12% and − 1.7% (Table 3), thus demonstration of an inspiration-related increase in normally aerated volume fraction represents an actual physiological change rather than artefact. Similarly, our results suggest that any inspiration-related decrease in CT density, blood volume or atelectatic volume fraction, and inspiration-related increases in blood volume above 4.7%, or overdistended volume fraction of more than 0.7% are not due to artefact.

A partial explanation for the improved matching between dCT and whole-lung variables in inspiration is evidenced by examining the effects inspiration has upon the slice. We demonstrated that the part of the lung that comprises the slice in expiration moves caudally with inspiration. Approximately 32% of the slice moved more than 5 mm in inspiration with the majority of this movement occurring within the middle third of the lung following injury, or within the middle and dependent thirds in uninjured animals (Fig. 5). The amount that moved more than 10 mm was minimal. It can be expected, therefore, that the part of the lung imaged in dCT during inspiration comprises two thirds of the slice imaged during expiration and one third of the next 5-mm slice above this.

Our CT scanner was able to simultaneously acquire three adjacent 5 mm slices during dCT. Thus, we were able to determine the effects this inspiratory movement would have had upon the make-up of the imaged slice. We demonstrated a small effect of cranio-caudal distance upon CT density, gas and normally aerated volume fractions, with the former increasing in the cranial direction (i. e. in the direction of the hila) and the latter two decreasing. This effect of cranio-caudal level upon CT density was not demonstrated in a previous study using 8-mm slice separation [10]; however, this other study assessed the difference based upon slices selected from whole-lung spiral CT during end-expiratory and end-inspiratory apnoeas. These slices may have differing compositions from dCT slices as end-inspiratory apnoeas are typically longer than the 4 s required for lung recruitment in the saline-lavage model [4], whereas inspiratory time in our dCT imaging was only 2 s. The movement of denser slices located closer to the hila into the field-of-view of the CT scanner during inspiration would cause an increase in density of the dCT slice imaged (prior to considering the effects of an increased gas volume during inspiration). This is in keeping with the underestimation of whole-lung CT density by dCT in expiration being reduced in inspiration. Similar effects are seen with the other variables.

### Limitations of this study

The main limitation of this study is that the use of whole-lung imaging during apnoeas as the gold-standard may not be appropriate and that four-dimensional imaging of the entire lung during tidal ventilation, as previously described using modification of the ECG-gating procedure used for cardiac CT [24], may be a better comparator, but such technology was unavailable for the DECT imaging in our scanner. Of course, if such technology were routinely available, it would obviate the need for using the single-slice technique to study experimental lung injury.

The finding that the single-slice technique is representative of the whole lung in uninjured and lung-lavaged pigs is not necessarily directly applicable to other ventilatory techniques (e.g. airway pressure release ventilation [25] or proning), lung injury models or human studies of ARDS. No animal model is capable of reproducing all of the key characteristics of ARDS in humans and any animal model is relevant for only limited aspects of ARDS pathophysiology in humans. In particular, in the case of our study, the anatomy of the pig thorax is such that a large volume of lung parenchyma exists between the inferior border of the heart and the diaphragm, which is suitable for use with the single-slice technique. This region contains representation of four out of the six pig lung lobes as previously characterised [26]. In the human, the heart sits upon the diaphragm and no such juxtadiaphragmatic slice exists that would be a suitable candidate to accurately represent the entire lung. In the case of normal human lungs and ARDS patients, a technique using 10 separate levels and extrapolating them to the entire lung may be more appropriate [27].

Finally, the choice of level of the slice used in dCT imaging is important, as well as the segmentation used to exclude regions outside the lung parenchyma. We pragmatically chose a region of the lung that contains the largest amount of lung within the 5-mm slice and demonstrated this was representative of the whole lung. Other studies [10, 27, 28] have assessed slices taken at different levels along the cranio-caudal axis, and thus, direct comparisons may not be appropriate. The hilar vessels and bronchi contain a large proportion of very high- and very low-density voxels, respectively, and inclusion of a small amount of these regions can dramatically affect results. Previously, it has been demonstrated that such regions comprise about 6% of lung volume segmented for dCT vs whole-lung studies [9], and thus, again, any small changes in what is chosen as comprising the segmented area or not would lead to large discrepancies between reported volumes in different studies.

## Conclusions

Dynamic CT images of a juxtadiaphragmatic slice of lungs are representative of the whole lung in the saline-lavage lung injury model and in pigs with uninjured lungs. This agreement is valid for classical single-energy parameters including CT density and following segmentation into fractions dependent upon lung density, as well as dual-energy CT indices following iodine contrast administration. Inspiration does cause a small change in the relationship due to denser regions nearer the hilum moving into the slice during inspiration. Authors of studies reporting tidal variations in parameters consistent with a decrease in CT density and atelectasis during inspiration can be reassured these are outside the limits of error introduced by the dCT technique and therefore represent true (patho)physiology. Overall, these results are confirmatory of previous findings [9, 10], extend the agreement to DECT indices and further explore the effects of inspiration with morphological analysis.

## Supplementary information

**Additional file 1.** Supplementary Methods, Results and Discussion. **Table S1.** Effects of injury, inspiration, PEEP and VT on the bias between dCT and whole-lung means. **Table S2.** Effects of injury, inspiration, PEEP and VT on correlation between whole-lung and dCT density distributions. **Figure S1.** Effects of PEEP and tidal volume upon the discrepancy between dynamic CT and whole-lung CT mean values for the eight studied variables. **Figure S2.** Distributions of correlation coefficients between whole-lung and dCT variables.

## Data Availability

The datasets generated and/or analysed during the current study are not publicly available due to the size of the source CT image files and lack of use for any other purpose than the current study in which they are summarised, but they are available from the corresponding author on reasonable request. The dual-energy CT three-material differentiation algorithm described here is available under a MIT licence from 10.5281/zenodo.3346937.

## Abbreviations

ARDS: Acute respiratory distress syndrome
CT: Computed tomography
dCT: Dynamic computed tomography
DECT: Dual-energy computed tomography
FiO_2_: Inspired oxygen concentration
HU: Hounsfield Unit
IQR: Inter-quartile range
PaO_2_: Arterial partial pressure of oxygen
PEEP: Positive end-expiratory pressure
SD: Standard deviation
