# Supplementary material for: Dynamic single-slice CT estimates whole-lung dual-energy CT variables in pigs with and without experimental lung injury
Source: Intensive Care Med Exp. 2019 Nov 1;7:59. doi: 10.1186/s40635-019-0273-y (PMC6825104; doi:10.1186/s40635-019-0273-y)

# **Dynamic single-slice CT estimates whole-lung dual-energy CT variables in pigs with and without experimental lung injury**

John N Cronin, João Batista Borges, Douglas C Crockett, Andrew D Farmery, Göran Hedenstierna, Anders Larsson, Minh C Tran, Luigi Camporota and Federico Formenti

Supplementary Data File

Supplementary Methods……………………………….……………2

Supplementary Results………………….…………………………..6

Supplementary Discussion……………….…………………………7

Supplementary References……………………………….................9

Table S1…………………………………………………………….10

Table S2…………………………………………………………….11

Figure S1…………………………………………………...............12

Figure S2…………………………………………………...............13

## Supplementary Methods

### Animal preparation and instrumentation

Eight domestic pigs (mean weight 31.1 kg (SD 1.5 kg)) sourced from a local farm were studied under general anaesthesia with mandatory-mode mechanical ventilation provided via tracheostomy. The animals were premedicated with intramuscular xylazine 2 mg/kg, ketamine 20 mg/kg and midazolam 0.5 mg/kg following which an ear vein was cannulated and intravenous ketamine 32 mg/kg/hr, fentanyl 4 mcg/kg/hr and midazolam 0.16 mg/kg/hr commenced. Adequacy of anaesthesia was confirmed by absence of reaction to painful stimulation between the front hooves, and absence of any signs of sympathetic stimulation after paralysis. Muscle relaxation was achieved using continuous infusion of rocuronium titrated against spontaneous respiratory effort. Normovolaemia was maintained with intravenous infusion of Ringer’s lactate solution at 20 mL/kg/hr for the first hour followed by 10 mL/kg/hr.

Mechanical ventilation was performed using a Servo-I ventilator (Maquet, Rastatt, Germany) with the animals in dorsal recumbency. During the instrumentation phase, ventilation was provided in volume control mode with tidal volumes (V_T_) of 10 mL/kg, respiratory rate (RR) 25 breaths/min, inspiratory:expiratory (I:E) ratio 1:2 and FiO_2_ 0.3. ECG, invasive systemic, central venous and pulmonary artery blood pressures were transduced using a standard clinical monitor (IntelliVue M8004A, Philips Healthcare, Best, Netherlands). Digital outputs from the ventilator and clinical monitor were continuously recorded using the acqIS software (EPiQ Life Science AB, Kista, Sweden). Pulmonary artery flotation catheter thermodilution cardiac output measurements and arterial blood gas analyses were performed at the beginning and end of the scanning series for each animal. At the end of the experimental protocol animals were killed under anaesthesia with an overdose of potassium chloride.

### CT scan sequence

All CT images were collected with a Somatom Definition Flash dual-source CT scanner (Siemens, Erlangen, Germany). First, high-spatial resolution single energy CT images were acquired during apnoeas to facilitate the morphological analyses of the lung. PEEP was titrated from 0 cmH_2_O to 20 cmH_2_O in steps of 5 cmH_2_O. At each PEEP level, mechanical ventilation was performed at RR 12 breaths/min in volume control mode with V_T_ 10 mL/kg for two minutes. At the end of this period, a spiral CT scan of the entire thorax was performed during an end-expiratory pause, followed by delivery of a single inspiratory breath and a second scan during an end-inspiratory pause. Airway pressure was maintained during pauses using a constant bias flow function available in the ventilator. Each single energy scan was performed using 120 kVp tube voltage, voxel size 0.5x0.5x0.6 mm and 64 scans per revolution.

Following the single energy series, dynamic and static DECT images were obtained with the addition of intravenous iodine contrast. Ventilation was performed in pressure control mode, RR 10 breaths/min, I:E 1:2, 0% ramp time such that dynamic scans obtained at 1 Hz frequency would acquire 4 frames in expiration and 2 in inspiration. Pressure control ventilation was utilised in this part of the study to reduce the chance of the first of two inspiratory frames being half-way between expiration and inspiration and thus requiring exclusion from further analysis. All combinations of PEEP 5, 8, 10 and 12 cmH_2_O and V_T_ target 7, 10 and 15 mL/kg were studied in each animal in a randomised order for a total of 12 ventilatory conditions per animal. After two minutes of ventilation in each condition, constant-rate iodine infusion (iomeprol; Iomeron 400 mg/mL, Bracco Imaging Scandinavia AB, Göteborg, Sweden) was commenced into the superior vena cava at a rate of 300 mg/s for 35 seconds. 16 seconds into the infusion, dynamic dual-energy CT scans were acquired of three 0.5x0.5x5 mm juxtadiaphragmatic slices at 1 Hz intervals for 19 seconds. The iodine protocol was selected to aim for a constant target pulmonary blood iodine concentration of approximately 4 mg/mL assuming a cardiac output of 4.5 L/min (similar to that seen in this study), and the scan period sufficient to image two complete breaths per ventilatory condition, but not so long that significant volumes of iodine would be seen to recirculate. The delay between the start of the infusion and the start of the scan allowed the portion of the descending thoracic aorta visualised in the slice to be opacified. The particular slice chosen for analysis was the most caudal of the three dynamic slices, which was positioned approximately mid-way between the carina and diaphragm in a region containing the greatest antero-posterior lung dimension and contained regions of the left and right caudal lobes and the right middle and accessory lobes. This slice shows the apex of the heart and the intra-thoracic inferior vena cava approximately one-half to two-thirds of the way from the anterior to the posterior surface of the lung. An example of the chosen slice is provided in the main manuscript in Fig. 1.

After acquisition of the dynamic series in each ventilatory condition, spiral volume DECT scans were obtained during an end-expiratory pause, followed by a single inspiratory breath and then again during an end-inspiratory pause. Voxel size was 0.5x0.5x5 mm for each scan. For all DECT images tube voltages were 80 and 140 kVp for the two energy levels.

### Statistical analyses

Eight variables of interest were chosen to determine the agreement between dCT and whole-lung images. These were CT density of the merge volume, and volume fractions of soft tissue, gas, iodinated blood as well as atelectatic, poorly aerated, normally aerated and overdistended regions. Mean values of each of these variables between dCT and whole-lung values were investigated with linear correlation, Bland-Altman analysis and paired t-test. The variation in discrepancy between dCT and whole-lung values was investigated using a four-way analysis of variance with presence of injury, inspiration-vs-expiration, PEEP and V_T_ as factors, employing Tukey’s post-hoc test as appropriate. The 95% limits of intra-tidal variation in bias between dCT and whole-lung were calculated as mean ± 1.96xSD. Normality was confirmed by examination of the relevant histograms, QQ plots and application of the Kolmogorov-Smirnov test. Correlation between distributions of merge volume CT densities as well as soft tissue, gas and iodinated blood volume fractions was assessed using Pearson’s product-moment correlation coefficient. Correlation coefficients between distributions were transformed via the function

$$x_{i}^{*}= \frac{1}{2} log \frac{x_{i}}{1 - x_{i}}$$

(where x_i_ is an input value and x_i_^*^ the corresponding transformed value) to achieve normality [1] prior to further investigation of the effects of injury, inspiration/expiration, PEEP and V_T_ with analysis of variance. To investigate the effect of cranio-caudal position within the three concurrently acquired dCT slices, the mean variation in the middle and cranial slice compared with the caudal slice was determined for each variable of interest and linear regression employed to determine the effect of a 1 mm change in position upon each particular variable.

## Supplementary Results

### Effect of PEEP and Tidal Volume on bias and correlation between dCT and whole-lung values

The effects of injury, inspiration, PEEP and V_T_ upon the difference between dCT and whole-lung variables was further investigated using four-way analysis of variance. Detailed comparison results are provided in Supplementary Table 1, with the effects of PEEP and tidal volume summarised in Supplementary Figure 1.

### Effects of injury, inspiration, PEEP and VT upon correlation between dCT and whole-lung density distributions

Correlation between dCT and whole-lung density distributions was overall good with median *r*=0.93 (IQR 0.09; Supplementary Figure 2a). The distribution of these points was non-normal (*P*<0.001) however normality was improved following normalization transform (*P*=0.22; Supplementary Figure 2b). The greatest effect size was seen with the effect of inspiration upon CT density, gas and iodinated blood volume fractions, as well as the effect of PEEP upon gas volume fractions (Supplementary Table 2). Statistically significant (*P*<0.05) effects were seen in other variables however the effect size was small. There was a significant interaction between inspiration and tidal volume for matching of CT density (*P*=0.02) and gas volume fraction (*P*=0.02). In both cases, the effect of an increase in tidal volume was to augment the increase in correlation seen with inspiration. No interaction was seen between PEEP and inspiration.

## Supplementary Discussion

### Effects of PEEP and tidal volume upon matching between dCT and whole-lung variables

The overall effect seen in the main results of this paper is that inspiration has the greatest effect upon the matching between dCT and whole-lung variables. Inspiration leads to caudal displacement of the studied slice and an increase in matching in mean CT densities, fractional volumes and fractions by mass of all subregions (atelectasis, poor aeration, normal aeration and overdistension). It is therefore not surprising that an increase in tidal volumes augments this matching by presumably increasing inspiration-related caudal displacement of the slice, whereas the effect of PEEP is to increase the matching independent of inspiration.

We demonstrated increased caudal displacement of the imaged slice at PEEP 10 cmH_2_O compared with 0 and 20 cmH_2_O. We did not investigate the causes behind this further, but can speculate that the reduced displacement seen at PEEP 0 cmH_2_O represents poor ventilation of the imaged slice (and preferential ventilation of non-imaged regions) in the setting of atelectasis. The reduced displacement at PEEP 20 cmH_2_O probably represents the decreased specific ventilation seen when the same tidal volume is applied to a lung with an increased end-expiratory lung volume. The change in the area of greatest displacement away from the dependent region and towards the middle of the lung in lung injury (Fig. 6c) is consistent with the reduced compliance of the dependent half of the lung as measured by electrical impedance tomography in experimental lung injury [2].

### Other limitations

Overall, we have demonstrated that the dCT slice closely approximates with a small bias the make-up of the whole lung in terms of eight separate variables of interest. We have defined the gold-standard measurement as the make-up of the whole lung during apnoeas at end-inspiration and end-expiration, however this may not be appropriate for the types of studies that use dCT, because of differences related to dynamics of lung collapse and recruitment. Typically, dCT is employed to assess the changes in lung aeration throughout the respiratory cycle [3-6], and the physiology of the lung during inspiration in tidal ventilation is possibly different to that of the lung during a prolonged inspiratory pause such as was required to perform the whole-lung CT scans used in this study. The majority of recruitment takes around 4 s to occur in the saline-lavage lung injury model [7], however our inspiratory time was only 2 s. We demonstrate that whole-lung CT had a greater fractional volume of atelectasis than dCT, with this excess reducing in inspiration. It is possible this reduction is due to the prolonged inspiratory pressures required to perform the apnoea for our whole-lung images, rather than a cyclical change in the accuracy of dCT. Ideally, we would like to be able to perform 4-dimensional DECT imaging of the entire thorax during tidal ventilation, but this is limited by the currently available technology.

A limitation of the morphological analysis technique used in this study is that it expects the deformation map to be continuous within the three-dimensional image and representable by a series of B-spline curves. During ventilation, the lung slides along the pleura such that the deformation at the pleural interface would be better represented by a step-change in these curves. We did not detect an obvious difference between the fixed and registered moving volumes on manual inspection in our data and aimed to exclude this pleural-edge artefact from our results by dividing the lung into twelve regions along the gravity vector such that only the first and last would contain a relatively large proportion of pleural edge (Fig. 5b).

## Supplementary References

1. Pituch KA, Stevens J (2016) Applied multivariate statistics for the social sciences. Routledge, Abingdon, UK

2. van Genderingen HR, van Vught AJ, Jansen JRC, (2003) Estimation of regional lung volume changes by electrical impedance pressures tomography during a pressure-volume maneuver. Intensive Care Med 29: 233-240

3. David M, Karmrodt J, Bletz C, David S, Herweling A, Kauczor HU, Markstaller K, (2005) Analysis of atelectasis, ventilated, and hyperinflated lung during mechanical ventilation by dynamic CT. Chest 128: 3757-3770

4. Markstaller K, Kauczor HU, Weiler N, Karmrodt J, Doebrich M, Ferrante M, Thelen M, Eberle B, (2003) Lung density distribution in dynamic CT correlates with oxygenation in ventilated pigs with lavage ARDS. Br J Anaesth 91: 699-708

5. Formenti F, Bommakanti N, Chen R, Cronin JN, McPeak H, Holopherne-Doran D, Hedenstierna G, Hahn CEW, Larsson A, Farmery AD, (2017) Respiratory oscillations in alveolar oxygen tension measured in arterial blood. Sci Rep 7: 7499

6. Crockett DC, Cronin JN, Bommakanti N, Chen R, Hahn CEW, Hedenstierna G, Larsson A, Farmery AD, Formenti F, (2019) Tidal changes in PaO2 and their relationship to cyclical lung recruitment/derecruitment in a porcine lung injury model. Br J Anaesth 122: 277-285

7. Neumann P, Berglund JE, Mondéjar EF, Magnusson A, Hedenstierna G, (1998) Dynamics of lung collapse and recruitment during prolonged breathing in porcine lung injury. J Appl Physiol 85: 1533-1543

## Table S1. Effects of injury, inspiration, PEEP and V_T_ on the bias between dCT and whole-lung means.

| **Variable** | **Injured vs Uninjured** | **Inspiration vs Expiration** | **PEEP**  **(12 *vs* 5 cmH_2_O)** | **Tidal Volume**  **(15 *vs* 7 mL/kg)** |
| --- | --- | --- | --- | --- |
| CT density (HU) | -25 (33) *vs* -38 (30); *P*<0.001 | -12 (24) *vs* -51 (26); *P*<0.001 | -17 (26) *vs* -50 (28); *P*<0.001 | -24 (36) *vs* -40 (26); *P*<0.001 |
| FV-soft tissue (%) | 0.2 (3.1) *vs* -2.5 (2.4); *P*<0.001 | -0.7 (2.7) *vs* -1.7 (3.4); *P*=0.013 | *ns*  *P*=0.20 | *ns*  *P*=0.73 |
| FV-gas (%) | 3.5 (3.0) *vs* 4.9 (2.7); *P*<0.001 | 2.4 (2.2) *vs* 6.1 (2.4); *P*<0.001 | 3.0 (2.5) *vs* 5.9 (2.6); *P*<0.001 | 3.6 (3.3) *vs* 4.9 (2.4); *P*<0.001 |
| FV-iodinated blood (%) | -3.7 (2.8) *vs* -2.4 (2.9); *P*<0.001 | -1.7 (2.3) *vs* -4.4 (2.9); *P*<0.001 | -1.8 (2.5) *vs* 4.0 (2.8); *P*<0.001 | -2.4 (2.8) *vs* -3.4 (2.4); *P*=0.037 |
| FV-atelectasis (%) | *ns*  *P*=0.12 | -1.4 (2.5) *vs* -4.1 (3.2); *P*<0.001 | -1.3 (2.6) *vs* -4.8 (3.3); *P*<0.001 | -2.3 (3.4) *vs* -3.4 (3.0); *P*=0.038 |
| FV-poorly aerated (%) | -3.0 (3.6) *vs* -5.7 (3.5); *P*<0.001 | -2.3 (2.9) *vs* -6.4 (3.5); *P*<0.001 | *ns*  *P*=0.051 | *ns*  *P*=0.29 |
| FV-normally aerated (%) | 6.1 (5.4) *vs* 9.3 (4.9); *P*<0.001 | 4.2 (3.8) *vs* 11.1 (4.4); *P*<0.001 | 5.4 (4.6) *vs* 10.7 (4.9); *P*<0.001 | 6.9 (6.1) *vs* 8.8 (4.5); *P*=0.002 |
| FV-overdistended (%) | -0.6 (0.4) *vs* -0.5 (0.2); *P*=0.001 | -0.5 (0.3) *vs* -0.7 (0.2); *P*<0.001 | -0.4 (0.3) *vs* -0.7 (0.3); *P*<0.001 | *ns*  *P*=0.58 |

Effects of injury, inspiration, PEEP and tidal volume upon the discrepancy between dCT and whole-lung means of eight different variables. Data are represented as mean discrepancy between dCT and whole-lung mean (SD) in first condition (e.g. injured) *vs* mean discrepancy (SD) in second condition (e. g. uninjured) where *P*<0.05 in the original ANOVA. For PEEP and tidal volume, two comparisons from the *post-hoc* testing are provided for comparison – these represent the groups with the largest effect upon mean discrepancy and the choice of these groups was consistent for all analyses. A negative discrepancy means dCT underestimated that variable with respect to whole-lung. The largest magnitude of effect was consistently with inspiration *vs* expiration. FV – volume fraction; HU – Hounsfield Units; *ns* – not significant in original ANOVA (*P*>0.05) therefore *post-hoc* testing and magnitude of effect not calculated.

## Table S2. Effects of injury, inspiration, PEEP and V_T_ on correlation between whole-lung and dCT density distributions.

| **Variable** | **Uninjured** | **Injured** |  |  | ***P*** |
| --- | --- | --- | --- | --- | --- |
| CT density | 0.94 (0.06) | 0.92 (0.08) |  |  | 0.002 |
| FV-soft tissue | 0.98 (0.02) | 0.97 (0.04) |  |  | <0.001 |
| FV-gas | 0.88 (0.13) | 0.89 (0.10) |  |  | 0.49 |
| FV-iodinated blood | 0.90 (0.09) | 0.91 (0.10) |  |  | 0.30 |
|  | **Expiration** | **Inspiration** |  |  |  |
| CT density | 0.89 (0.08) | 0.95 (0.04) |  |  | <0.001 |
| FV-soft tissue | 0.97 (0.03) | 0.98 (0.02) |  |  | 0.01 |
| FV-gas | 0.84 (0.16) | 0.93 (0.07) |  |  | <0.001 |
| FV-iodinated blood | 0.88 (0.11) | 0.94 (0.06) |  |  | <0.001 |
|  | **PEEP 5 cmH_2_O** | **PEEP 8 cmH_2_O** | **PEEP 10 cmH_2_O** | **PEEP 12 cmH_2_O** |  |
| CT density | 0.89 (0.12) | 0.91 (0.08) | 0.93 (0.05) | 0.94 (0.04) | <0.001 |
| FV-soft tissue | 0.97 (0.03) | 0.98 (0.02) | 0.97 (0.03) | 0.98 (0.02) | 0.59 |
| FV-gas | 0.86 (0.18) | 0.88 (0.16) | 0.89 (0.11) | 0.91 (0.09) | <0.001 |
| FV-iodinated blood | 0.89 (0.11) | 0.91 (0.08) | 0.91 (0.08) | 0.93 (0.08) | 0.26 |
|  | **V_T_ 7 mL/kg** | **V_T_ 10 mL/kg** | **V_T_ 15 mL/kg** |  |  |
| CT density | 0.92 (0.07) | 0.93 (0.06) | 0.94 (0.07) |  | 0.006 |
| FV-soft tissue | 0.98 (0.02) | 0.97 (0.03) | 0.98 (0.03) |  | 0.13 |
| FV-gas | 0.88 (0.12) | 0.89 (0.11) | 0.89 (0.12) |  | 0.18 |
| FV-iodinated blood | 0.91 (0.11) | 0.90 (0.07) | 0.92 (0.09) |  | 0.009 |

Spearman’s product-moment correlation coefficients for correlation between whole-lung and dCT density distributions of four variables, and the effects of injury, inspiration, PEEP and tidal volume (V_T_) upon these. Data expressed as median (IQR) due to non-normality of correlation coefficient distributions, and *P* values represent the results of ANOVA upon correlation coefficients after a normalization transform. FV – volume fraction.

## Figure S1. Effects of PEEP and tidal volume upon the discrepancy between dynamic CT and whole-lung CT mean values for the eight studied variables. a) Mean lung density, b) three material differentiation results, and c) following segmentation based upon gas density. Soft tissue, poorly aerated fraction and overdistended fraction demonstrated minimal dependence upon PEEP and tidal volume, however in the other variables there was an increased agreement (points closer to the dotted line of no difference) with higher PEEP and higher tidal volumes. Points and error bars represent mean and SD of *n*=96 ventilatory conditions across 8 animals.


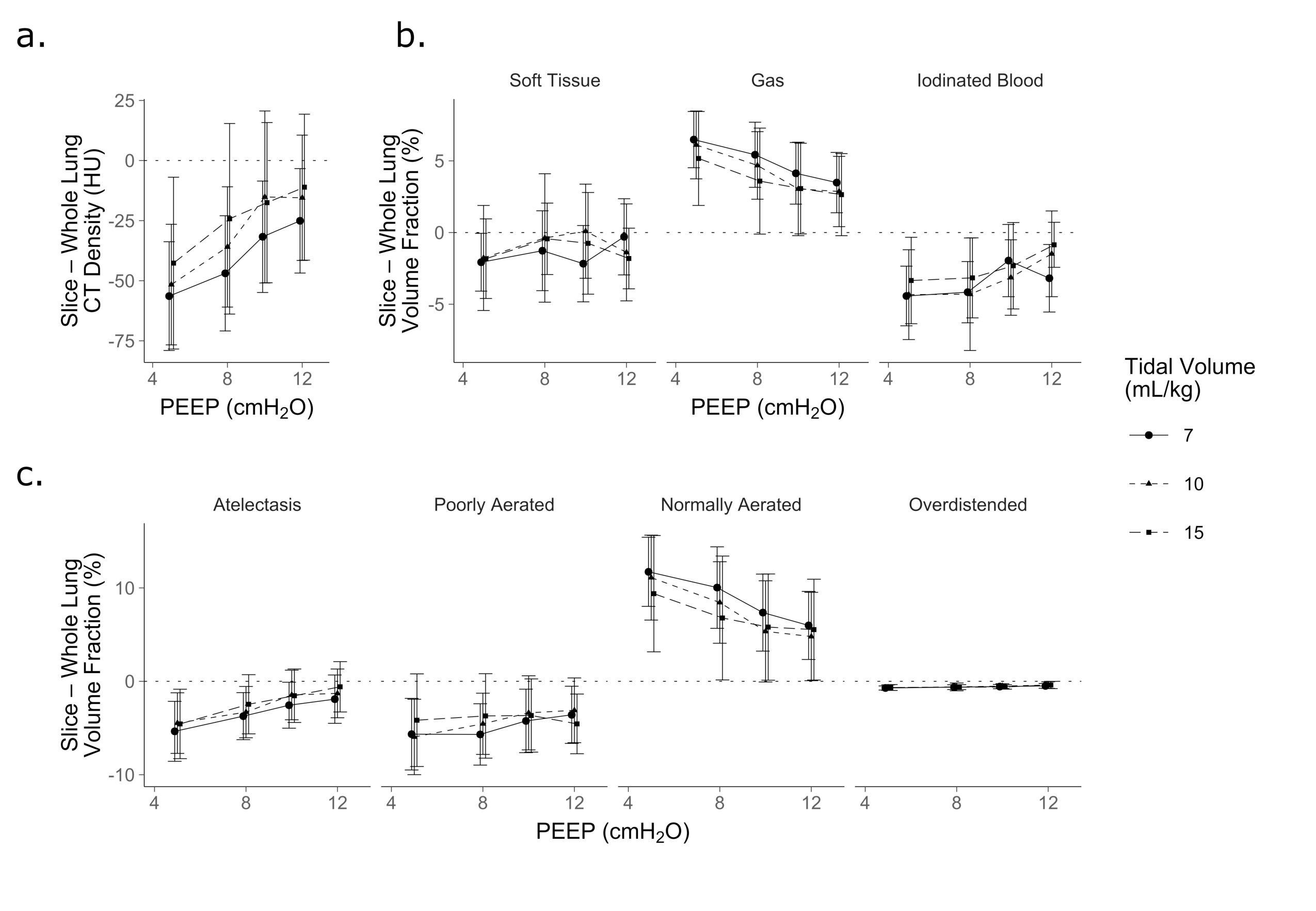


## Figure S2. Distributions of correlation coefficients between whole-lung and dCT variables. a) raw Pearson product-moment correlation coefficients demonstrating negatively skewed distribution with 66% of data points ≥ 0.9, b) correlation coefficients after normalization transform as described in Supplementary Methods with superimposed normal distribution for comparison. *n*=768, representing four variables (CT density, soft tissue, gas and iodinated blood volume fractions), four PEEP settings and three tidal volumes per each of 8 animals, with each correlation being performed in both inspiration and expiration.


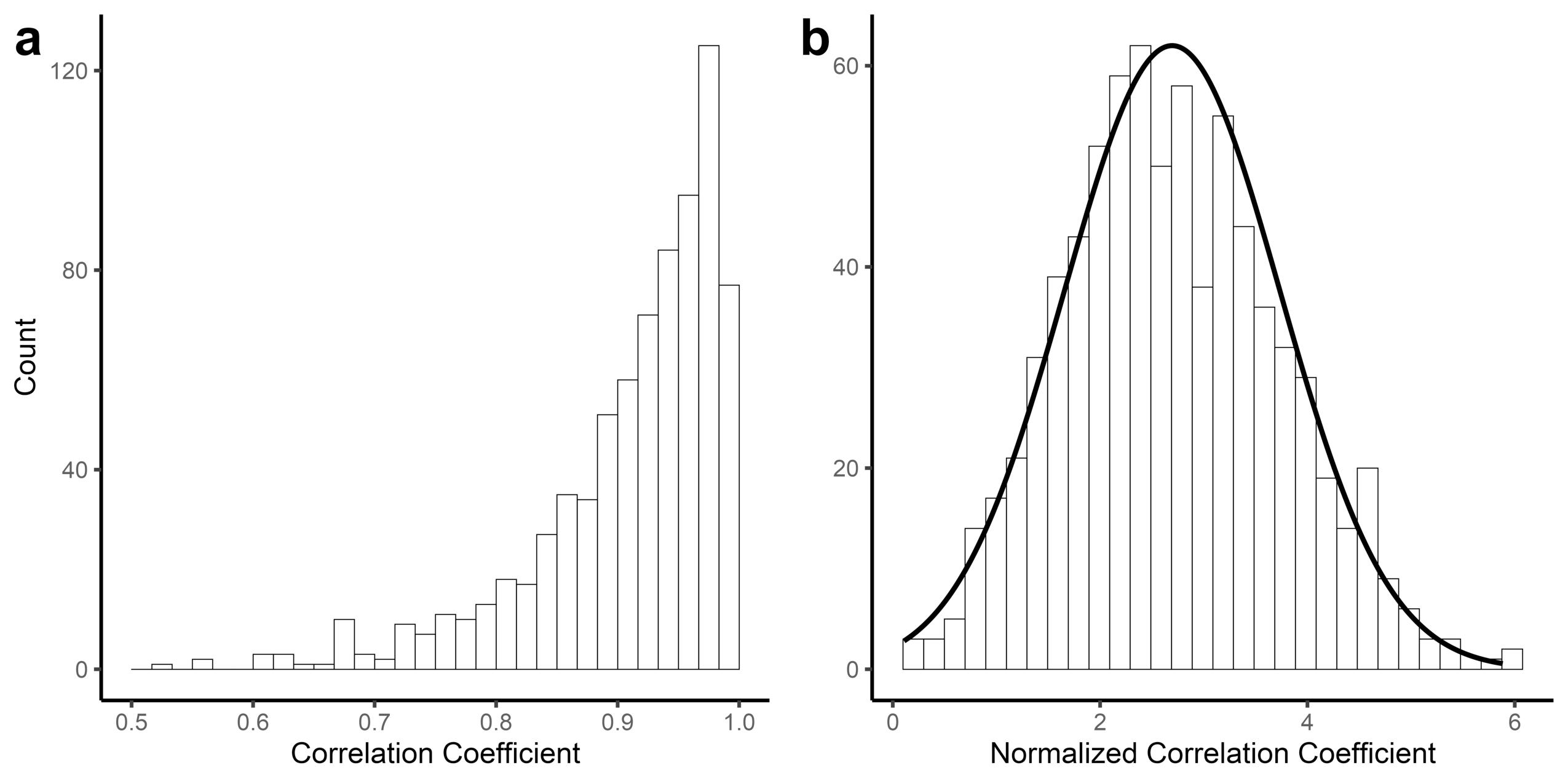

Supplement: Supplementary file 1 — Additional file 1. Supplementary Methods, Results and Discussion. Table S1. Effects of injury, inspiration, PEEP and VT on the bias between dCT and whole-lung means. Table S2. Effects of injury, inspiration, PEEP and VT on correlation between whole-lung and dCT density distributions. Figure S1. Effects of PEEP and tidal volume upon the discrepancy between dynamic CT and whole-lung CT mean values for the eight studied variables. Figure S2. Distributions of correlation coefficients between whole-lung and dCT variables. [file 40635_2019_273_MOESM1_ESM.docx]
